# Overcoming absolute dysphagia in a thirty-year-old patient with advanced anaplastic lymphoma kinase-positive non-small cell lung cancer: a case report

**DOI:** 10.3389/fonc.2026.1840539

**Published:** 2026-05-28

**Authors:** Luca Carlofrancesco Ammoni, Giorgia Carola, Giuseppe Ippolito, Alice Baggi, Francesca Consoli, Andrea Esposito, Ilaria Pedrazzini, Alfredo Berruti, Salvatore Grisanti

**Affiliations:** Department of Medical and Surgical Specialties, Radiological Sciences and Public Health, Medical Oncology, University of Brescia, Azienda Socio-Sanitaria Territoriale (ASST) Spedali Civili, Brescia, Italy

**Keywords:** ALK translocation, dysphagia, enteral absorption, lorlatinib, non-small cell lung cancer

## Abstract

**Background:**

Anaplastic lymphoma kinase (ALK)-positive non-small cell lung cancer (NSCLC) accounts for approximately 3–7% of all NSCLCs and predominantly affects young, never-smoker patients. Lorlatinib, a third-generation ALK tyrosine kinase inhibitor (TKI), has demonstrated significant efficacy as first-line therapy. However, its exclusive oral formulation poses a challenge in patients with dysphagia.

**Case presentation:**

We report the case of a 30-year-old woman with metastatic ALK-positive NSCLC who presented with severe dysphagia due to bulky supraclavicular and laterocervical lymphadenopathy compressing the cervical oesophagus. Given the impossibility of swallowing, lorlatinib 100 mg daily was administered via a nasogastric tube after tablet crushing. Within days, rapid reduction of cervical lymph node swelling was observed, swallowing improved, and the patient resumed oral intake. Imaging performed two weeks later showed partial response; at six months, PET-CT demonstrated complete metabolic response.

**Discussion:**

This case supports the feasibility and effectiveness of lorlatinib administration via the enteral route in patients unable to swallow. Despite the lack of formal evidence for alternative formulations, pharmacokinetic data suggest adequate absorption.

**Conclusion:**

Crushed lorlatinib administered through a nasogastric tube represents a practical and effective option for dysphagic patients with ALK-positive NSCLC requiring early target-directed therapy.

## Background

Anaplastic lymphoma kinase (ALK)-positive lung cancers account for approximately 3-7% of non-small cell lung cancers (NSCLC), mainly concerning non-squamous subtypes and occurring predominantly in young, never-smoker patients (1). The role of chemotherapy is quite limited, and the use of immune checkpoint inhibitors (ICIs) has not shown meaningful efficacy in this context (2). From the approval of crizotinib onwards, the advent of ALK tyrosine-kinase inhibitors (TKIs) has dramatically changed the natural history of this aggressive disease (3–6).

Lorlatinib is a third-generation ALK-TKI that selectively targets anaplastic lymphoma kinase rearrangements. It binds to the ATP-binding site of this kinase, inhibiting its phosphorylation activity and thereby blocking downstream signaling pathways involved in cell proliferation and survival, such as the PI3K/AKT and MAPK pathways. This drug has been approved for the treatment of patients with advanced NSCLC with ALK rearrangement. Such approval in the first-line setting is based on the results of the CROWN study that demonstrated the superiority of lorlatinib over crizotinib in a population of treatment-naive patients, with an objective response rate (ORR) of 76% vs 58% and high intracranial activity (7). The 5-year update of the CROWN study confirmed the efficacy of lorlatinib with a 5-year progression-free survival (PFS) of 60% and 81% reduction in risk of progression compared to standard treatment (8). The recommended dose is 100 milligrams (mg) once daily, but in case of toxicities, dose reductions are allowed per protocol (mainly dysmetabolic or neurological/psychiatric adverse events). Oral administration has been required: tablets must be swallowed intact, without chewing or crushing (9). Currently, no evidence exists regarding alternative routes of lorlatinib administration, especially for patients with dysphagia who are unable to ingest the tablet whole.

Here we report the case of a young patient diagnosed with metastatic ALK-positive NSCLC presenting with bulky supraclavicular and laterocervical nodes causing complete dysphagia.

## Case report

**Figure 1:**
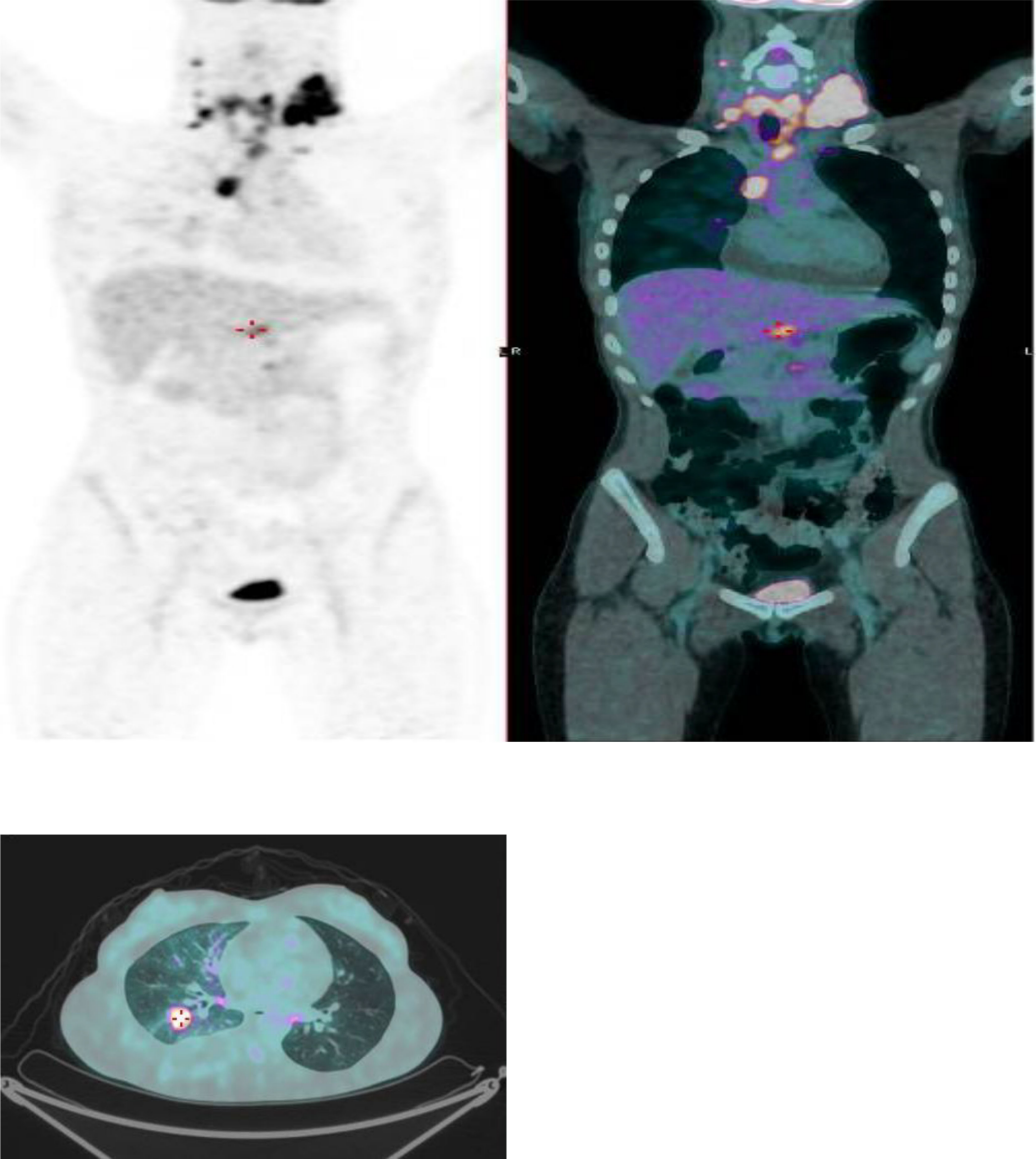
PET scan before lorlatinib.

In June 2025, a thirty-year-old never-smoker woman with no significant past medical history, no known occupational or environmental exposures and unremarkable social history went to the emergency room for recent onset of swelling in the supraclavicular fossa associated with progressive dysphagia, firstly to solids only and later to liquids too. On physical examination, multiple enlarged laterocervical and supraclavicular lymph nodes were palpable. Chest auscultation revealed reduced vesicular breath sounds at the right lung base. Routine laboratory tests were within normal limits. She was hospitalized and underwent a total body computed tomography (CT) scan with contrast medium and a positron emission tomography (PET) which showed a lung lesion in the right lower lobe, pleural and pericardial effusion, mediastinal, abdominal, supraclavicular and laterocervical bulky lymph nodes conditioning “ab-extrinseco” compression of the cervical oesophagus (**Figure 1**). The histological examination of a right supraclavicular lymph node revealed the presence of metastases of lung adenocarcinoma with programmed death ligand 1 (PD-L1) tumour proportion score (TPS) of 70%. Molecular profiling performed using next-generation sequencing (NGS), aimed at detecting clinically actionable molecular alterations (including those involving EGFR, KRAS, BRAF, ROS1, NTRK, MET, and RET), as well as mutations with prognostic relevance (such as those involving TP53), identified the presence of an echinoderm microtubule-associated protein-like 4 (EML4)–ALK fusion.

**Figure 2:**
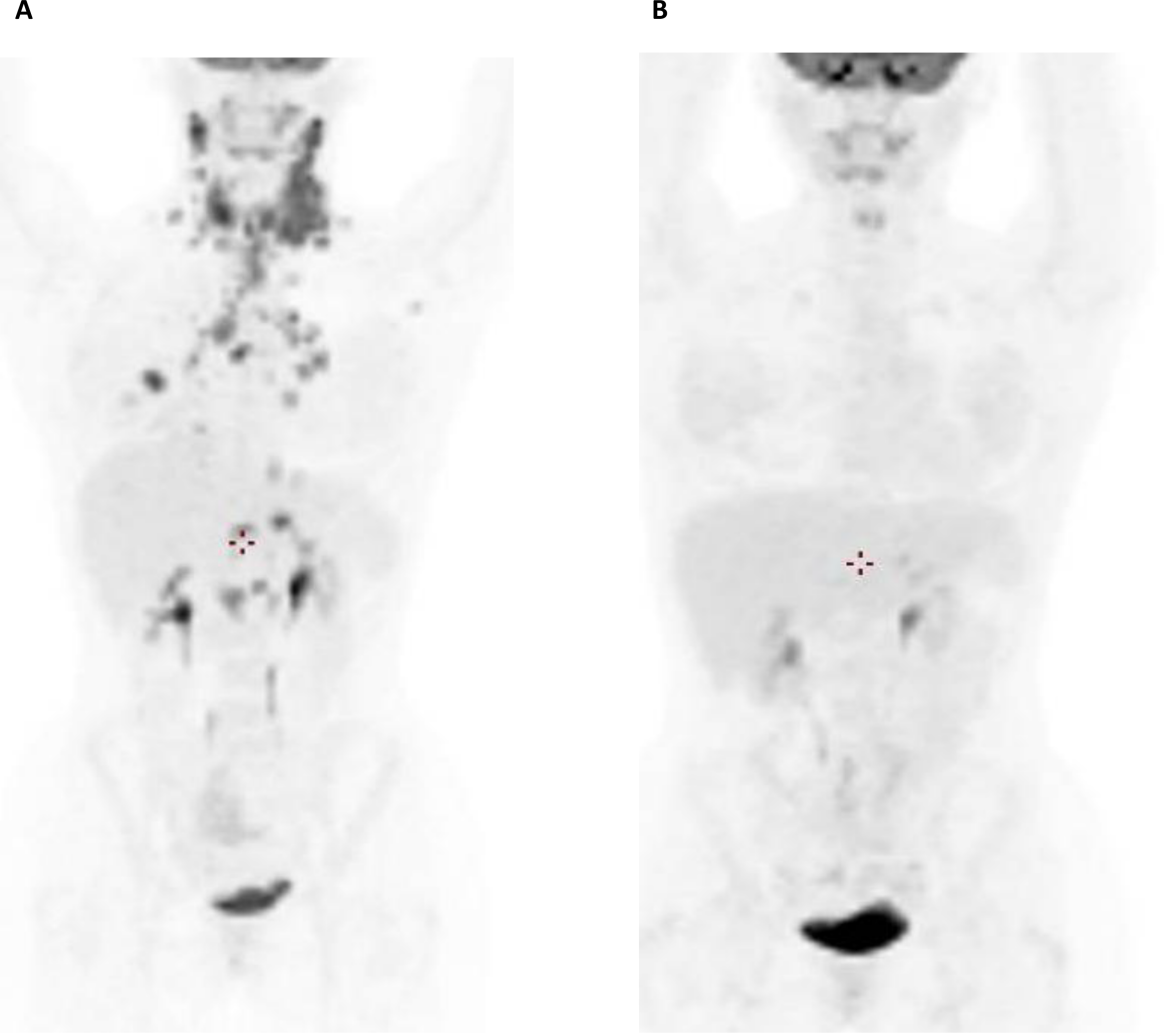
**(A)** PET scan before lorlatinib. **(B)** PET scan after 6 months of lorlatinib.

**Figure 3:**
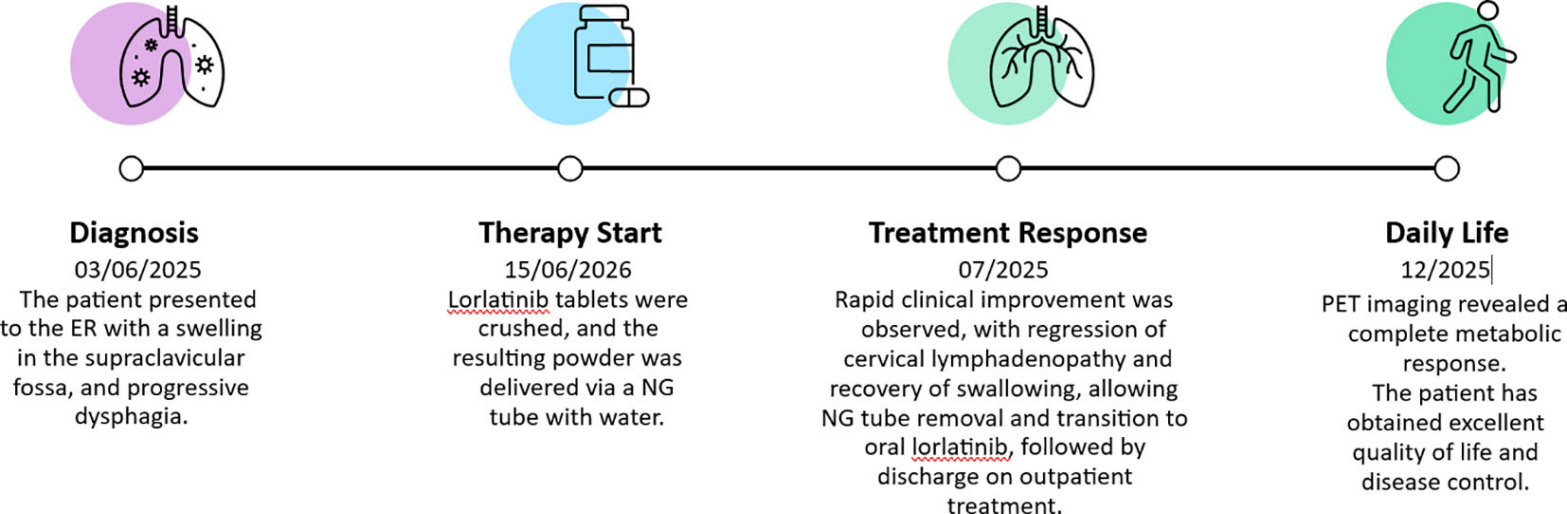
Timeline.

After multidisciplinary discussion, a first-line treatment with lorlatinib 100 mg daily was proposed to the patient. Given the absolute dysphagia, it was decided to place a nasogastric tube and administer the tablets of lorlatinib, once crushed, through this device. This “unconventional method” was chosen (over chemotherapy) with the aim of obtaining a shrinkage of the lymph node masses that could allow the future intake of the drug orally as required by the Summary of Product Characteristics. Within ten days the lymph nodes of the neck were reduced, swallowing was gradually restored, the nasogastric tube was removed, and the patient could return to oral feeding. The administration of lorlatinib was then changed to oral intake. Two weeks later CT scan was repeated, showing a partial response in all disease sites. The patient was therefore discharged and continued outpatient treatment with an excellent quality of life and disease control. After six months of therapy, a new PET showed complete metabolic response (**Figure 2**). Lorlatinib was well tolerated by the patient. The only reported adverse event was increased cholesterol levels, which required optimization of lipid-lowering therapy by the cardiologist. Because the patient is still on treatment, data regarding PFS and OS are not yet available, consistent with the findings of the CROWN study (8) (**Figure 3**).

## Discussion

Since the advent of ALK TKIs, the prognosis of patients with ALK-positive NSCLC has improved dramatically (3–7). This is particularly relevant given that these people are often young and active. The case presented here, concerning a thirty-year-old woman with diagnosis of ALK-positive NSCLC, focuses on the dysphagia caused by lymph node burden. It is a worsening symptom of quality of life as it compromises diet and medication. The major issue was represented by imminent visceral risks, both in terms of absolute dysphagia and airway compromise, making the choice of the first line treatment decisive. When choosing first-line therapy, immunotherapy was not considered given that the use of ICIs has not proved to be efficacious in this context, as emerged from data by the IMMUNOTARGET registry (2). This was a retrospective collection of oncogene-addicted NSCLC patients treated with PD-1/PD-L1 inhibitors in which the ALK-positive subgroup obtained an ORR of zero and a PFS of less than three months (2). Chemotherapy was excluded too because, although it could have been administered intravenously allowing to bypass absolute dysphagia, several trials clearly demonstrated the meaningful clinical benefit of ALK-inhibitors over chemotherapy and that this class of drugs performs better as first-line treatment. Indeed, ALK TKIs are the gold standard in this disease setting. They are commonly classified into first-, second-, and third-generation agents, with relevant differences in efficacy, central nervous system (CNS) penetration, and resistance profiles. The first evidence of ALK-TKIs superiority compared with traditional chemotherapy was provided by the global PROFILE 1014 phase III trial, in which crizotinib demonstrated a meaningful clinical benefit both in terms of response rate and survival (3). Time to response (TTR) was halved (1.4 vs 2.8 months), a finding which was further confirmed by the results from PROFILE 1019 in East Asian patients (10). However, crizotinib has poor CNS activity and development of resistance is inevitable (3). Second-generation TKIs, including alectinib (4), ceritinib (5) and brigatinib (6), showed improved systemic and intracranial efficacy compared to crizotinib and have long represented standard first-line options. However, resistance mechanisms, particularly ALK secondary mutations, inevitably emerge. Third-generation lorlatinib was designed to overcome a broad spectrum of resistance mutations and to achieve high CNS penetration. The results of the CROWN study showed lorlatinib impressive effectiveness in terms of PFS and brain activity (common site of metastasis in ALK-positive NSCLC) (8). For this class of drugs, however, only the oral formulation is recommended, which undoubtedly represented a big problem considering the absolute dysphagia. To date there are no data supporting forms of administration other than oral, nor is the absorption of the drug known when the tablets are crushed. In a phase I study by Hibma et al, the absolute bioavailability of oral lorlatinib was far better compared to IV administration (11). Furthermore, authors observed that the variability estimates for lorlatinib plasma exposure following IV dose versus oral dose were similar, suggesting that any further efforts to develop other formulations to improve absorption would not be of benefit (11). These findings had already emerged by *in vitro* studies demonstrating that lorlatinib is highly permeable, well absorbed and has a low first-pass effect (12, 13). In a preclinical pharmacokinetic study in mice, moreover, there was a higher distribution of lorlatinib in muscle tissues, indicating that the lipophilicity of lorlatinib was not as high as expected (14).

After extensive research, we found only two case reports of enteral lorlatinib in the literature (**Table 1**). The first was a treatment-naïve patient with carcinomatous meningitis, poor performance status and dysphagia (15). The second referred to a patient with disease hyperprogression after previous chemo-immunotherapy conditioning dysphagia (16). In both cases, after the tablets were shredded and dissolved in water, lorlatinib was administered through a nasogastric tube. The outcome of both patients was favourable, thanks to a rapid clinical response that allowed to restore adequate swallowing (15, 16). Despite the absence of robust data on the feasibility and efficacy of enteral lorlatinib administration, our experience suggests that this approach is viable in selected cases with imminent visceral risk when a rapid disease response is required. Albeit case reports represent the lowest level of evidence in evidence-based medicine, they play an important role in integrating information from clinical trials in order to optimize daily clinical practice (17).

**Table 1:** Comparison of previously reported cases and the present case of enteral lorlatinib administration in ALK-positive NSCLC patients with dysphagia.

Patient characteristics	Sasaki et al.	Wang et al.	Our patient
Age	73	49	30
Sex	Female	Male	Female
Country	Japan	China	Italy
Diagnostic test performed to discover ALK-rearrangement	Immunohistochemistry (IHC) and fluorescent *in situ* hybridization (FISH)	Next-generation sequencing (NGS)	Next-generation sequencing (NGS)
Disease stage	IVb	IVb	IVb
Cause of dysphagia	Unconsciousness due to carcinomatous meningitis	External pressure-associated oesophageal stenosis due to disease hyper-progression	Bulky lymph nodes conditioning “ab-extrinseco” compression of the cervical oesophagus
Previous treatments	Alectinib	Cisplatin + pemetrexed + pembrolizumab in first line, cisplatin + docetaxel + bevacizumab in second line)	Treatment-naive
Time to oral intake	Four weeks	Eighteen days	Ten days
PFS	16 months	Not available	Not reached
OS	16 months	Not available	Not reached

## Conclusion

The present case report showed that the administration of lorlatinib by enteral route is feasible and effective, thus representing a valid option for dysphagic patients with ALK-positive NSCLC.

We are aware that case reports are generally considered a low level of evidence; however, there remains a notable lack of data in the scientific literature regarding alternative methods of administering lorlatinib. Our literature search revealed only two relevant case reports to guide clinical decision-making. This work aims to provide practical insights to assist clinicians in managing drug administration in complex patient scenarios.

## Data Availability

The original contributions presented in the study are included in the article/Supplementary Material. Further inquiries can be directed to the corresponding author.

## References

[1] Takeuchi K Choi YL Soda M Inamura K Togashi Y Hatano S . Multiplex reverse transcription-PCR screening for EML4-ALK fusion transcripts. Clin Cancer Res. (2008) 14:6618–24. doi: 10.1158/1078-0432.ccr-08-1018. PMID: 18927303

[2] Mazieres J Drilon A Lusque A Mhanna L Cortot AB Mezquita L . Immune checkpoint inhibitors for patients with advanced lung cancer and oncogenic driver alterations: results from the IMMUNOTARGET registry. Ann Oncol. (2019) 30:1321–8. doi: 10.1093/annonc/mdz167. PMID: 31125062 PMC7389252

[3] Solomon BJ Mok T Kim DW Wu YL Nakagawa K Mekhail T . First-line crizotinib versus chemotherapy in ALK-positive lung cancer. N Engl J Med. (2015) 373:1582. doi: 10.1056/nejmoa1408440. PMID: 26466011

[4] Peters S Camidge DR Shaw AT Gadgeel S Ahn JS Kim DW . Alectinib versus crizotinib in untreated ALK-positive non-small-cell lung cancer. N Engl J Med. (2017) 377:829–38. doi: 10.1056/nejmoa1704795. PMID: 28586279

[5] Soria JC Tan DSW Chiari R Wu YL Paz-Ares L Wolf J . First-line ceritinib versus platinum-based chemotherapy in advanced ALK-rearranged non-small-cell lung cancer (ASCEND-4): a randomised, open-label, phase 3 study. Lancet. (2017) 389:908. doi: 10.1016/s0140-6736(17)30123-x. PMID: 28126333

[6] Camidge DR Kim HR Ahn MJ Yang JC Han JY Lee JS . Brigatinib versus crizotinib in ALK-positive non-small-cell lung cancer. N Engl J Med. (2018) 379:2027–39. doi: 10.1056/NEJMoa1810171 30280657

[7] Shaw AT Bauer TM de Marinis F Felip E Goto Y Liu G . First-line lorlatinib or crizotinib in advanced ALK-positive lung cancer. N Engl J Med. (2020) 383:2018–29. doi: 10.1016/j.lungcan.2022.11.004. PMID: 33207094

[8] Solomon BJ Liu G Felip E Mok TSK Soo RA Mazieres J . Lorlatinib versus crizotinib in patients with advanced ALK-positive non-small cell lung cancer: 5-year outcomes from the phase III CROWN study. J Clin Oncol. (2024) 42:3400–9. doi: 10.1200/jco.24.00581. PMID: 38819031 PMC11458101

[9] https://www.ema.europa.eu. Available online at: https://www.ema.europa.eu (Accessed August 9, 2025).

[10] Wu YL Lu S Lu Y Zhou J Shi YK Sriuranpong V . Results of PROFILE 1029, a phase III comparison of first-line crizotinib versus chemotherapy in East Asian patients with ALK-positive advanced non-small cell lung cancer. J Thorac Oncol. (2018) 13:1539–48. doi: 10.4143/crt.2017.280. PMID: 29966800

[11] Hibma JE O'Gorman M Nepal S Pawlak S Ginman K Pithavala YK . Evaluation of the absolute oral bioavailability of the anaplastic lymphoma kinase/c-ROS oncogene 1 kinase inhibitor lorlatinib in healthy participants. Cancer Chemother Pharmacol. (2022) 89:839. doi: 10.1007/s00280-021-04368-1. PMID: 35303141 PMC9135853

[12] Johnson TW Richardson PF Bailey S Brooun A Burke BJ Collins MR . Discovery of (10R)-7-amino-12-fluoro-2,10,16-trimethyl-15-oxo-10,15,16,17-tetrahydro-2H-8,4-(metheno)pyrazolo[4,3-h][2,5,11]-benzoxadiazacyclotetradecine-3-carbonitrile (PF-06463922), a macrocyclic inhibitor of anaplastic lymphoma kinase (ALK) and c-ros oncogene 1 (ROS1) with preclinical brain exposure and broad-spectrum potency against ALK-resistant mutations. J Med Chem. (2014) 57:4720–44. doi: 10.1021/jm500261q. PMID: 24819116

[13] El Darsa H Abdel-Rahman O Sangha R . Pharmacological and clinical properties of lorlatinib in the treatment of ALK-rearranged advanced non-small cell lung cancer. Expert Opin Pharmacother. (2020) 21:1547–54. doi: 10.1080/14656566.2020.1774552. PMID: 32511029

[14] Chen W Shi Y Qi S Zhou H Li C Jin D . Pharmacokinetic study and tissue distribution of lorlatinib in mouse serum and tissue samples by liquid chromatography-mass spectrometry. J Anal Methods Chem. (2019) 2019:7574369. doi: 10.1155/2019/7574369. PMID: 30949374 PMC6425379

[15] Sasaki K Yokota Y Isojima T Fujii M Hasui K Chen Y . Enteral lorlatinib after alectinib as a treatment option in anaplastic lymphoma kinase-positive non-small cell lung cancer with triple problems: carcinomatous meningitis, poor performance status, and dysphagia-a case report. Respirol Case Rep. (2021) 9:e00796. doi: 10.1002/rcr2.796. PMID: 34123384 PMC8173452

[16] Wang H Wu Z Shi G Zhou J Xiao Z . Enteral lorlatinib after immune hyperprogression as a treatment option for anaplastic lymphoma kinase‑positive non‑small cell lung cancer: A case report. Oncol Lett. (2023) 26:526. doi: 10.3892/ol.2023.14113. PMID: 38020308 PMC10644362

[17] Gilbar PJ Goldspiel BR . The continuing importance of oncology case reports. J Oncol Pharm Pract. (2021) 27:263–5. doi: 10.1177/1078155220988577. PMID: 33470175

